# Asymptomatic Extrahepatic Portal Venous Obstruction: A Case Report

**DOI:** 10.7759/cureus.64037

**Published:** 2024-07-07

**Authors:** Priscilla Johnson, Johnson WMS, Senthil N, Balaji Singh K, Koushik A K

**Affiliations:** 1 Physiology, Sri Ramachandra Medical College and Research Institute, Sri Ramachandra Institute of Higher Education and Research, Deemed to be University, Chennai, IND; 2 Anatomy, Sri Lalithambigai Medical College and Hospital, Chennai, IND; 3 General Medicine, Sri Ramachandra Medical College and Research Institute, Sri Ramachandra Institute of Higher Education and Research, Deemed to be University, Chennai, IND; 4 General Surgery, Sri Ramachandra Medical College and Research Institute, Sri Ramachandra Institute of Higher Education and Research, Deemed to be University, Chennai, IND; 5 Medical Gastroenterology, Sri Ramachandra Medical College and Research Institute, Sri Ramachandra Institute of Higher Education and Research, Deemed to be University, Chennai, IND

**Keywords:** “pancytopenia”, acute calculus cholecystitis, portal hypertension, oesophageal varices, extrahepatic portal venous obstruction

## Abstract

This report describes a case of extrahepatic portal venous obstruction (EHPVO) with esophageal varices that would have led to significant bleeding if left untreated or inadequately managed. A 56-year-old diabetic and hypothyroid female visited our medical outpatient clinic to be assessed for pancytopenia and easy fatiguability. She experienced acute calculus cholecystitis 24 years ago, which was treated with a partial cholecystectomy. The laboratory tests showed indications of hypersplenism, characterized by anemia, leucopenia, and thrombocytopenia. The MRI results showed signs of long-term blockage of the portal vein outside the liver, with the liver tissue seeming normal. The upper gastrointestinal endoscopy showed grade IV esophageal varices, gastroesophageal varices 1, fundal varices, isolated gastric varices 2, and antral varices. The patient was diagnosed with EHPVO, and banding was performed as a preventive measure against upper gastrointestinal bleeding. Additionally, she was treated using conservative management techniques such as beta blockers. Endoscopic treatment is the standard of care for treating acute varices, while beta blockers are given as a secondary preventive measure. Despite cholelithiasis being a cause and/or sequelae to portal venous thrombosis, a clear explanation has not been offered to this patient while taking consent for cholecystectomy or thereafter. EHPVO is not frequently detected, and there is still a dearth of appropriate guidelines for managing this illness, even though it is a frequent cause of acute calculus cholecystitis and upper gastrointestinal bleeding.

## Introduction

Globally, extrahepatic portal venous obstruction (EHPVO) is the second-most common cause of portal hypertension. Portal venous thrombosis (PVT), a condition characterized by the partial or complete blockage of blood flow in the portal vein, caused by a thrombus either in the main trunk of the portal vein, its intrahepatic branches, or the splenic or superior mesenteric veins, is one of the main predisposing factors for EHPVO. Studies have reported that PVT is responsible for extrahepatic portal hypertension in about 5%-10% of the Western world and 40% of the Indian subcontinent [1,2].The pooled prevalence of PVT in 21 Chinese studies was 20.2% (95% confidence interval (CI):15.3%-25.8%) and the pooled prevalence of PVT in 11 Japanese studies was 24.7% (95% CI: 12.9%-38.9%) [3]. Ogren et al. conducted an epidemiological study using 23,796 consecutive autopsies to determine the lifetime cumulative incidence of PVT in the general population of Southern Sweden. They found that the overall prevalence of PVT in compensated cirrhosis was approximately 1% in the Western general population. In most cases, PVT was found to be secondary to underlying liver disease or malignancy, with an odds ratio of 17 (95% CI: 11-26). Additionally, in 14% of cases, no specific cause for PVT was identified [4]. However, inherited prothrombotic defects were detected in 15% of patients with PVT in India [5], and 58.3% were found to have anticoagulant deficiencies in Pakistan [6]. A meta-analysis has revealed that the prevalence of myeloproliferative disorder and Janus kinase 2 (JAK2) mutations in patients with PVT was 31.5% and 27.7%, respectively [7]. In individuals with non-malignant non-cirrhotic PVT, the odds ratios were 50 for oral contraceptive usage, 7 for prothrombin gene mutation, 1.5 for factor V Leiden, 5, 3, and 1 for deficiencies in protein C, protein S, and antithrombin III, respectively [8].

There is insufficient evidence-based literature to support a specific management method for EHPVO. The primary objective is to minimize blood loss and provide a bypass to alleviate pressure. The efficacy of beta-blockers as a first-line preventive measure has not been well investigated. While Meso-Rex bypass and splenorenal shunts are commonly suggested for the treatment of extrahepatic portal vein obstruction, sclerotherapy is also considered an option [9]. However, as sclerotherapy has a seven-fold higher risk of rebleeding and a three-fold higher risk of treatment failure than shunt surgery, endoscopic banding should be the therapy of choice.

We report a case of portal hypertension caused by extrahepatic portal vein obstruction with esophageal and gastric varices. The patient was treated with banding and beta blockers.

## Case presentation

A 56-year-old female, who is known to be diabetic and hypothyroid, presented to our medicine outpatient department for evaluation of pancytopenia and easy fatiguability. She did not report any past medical history of fever, vomiting, abdominal pain, joint pain, abnormal bleeding, thrombotic episodes, petechiae, or ecchymosis.

On detailed evaluation of her past history, she was incidentally found to have pancytopenia when she was evaluated for acute cholecystitis with suspected common bile duct calculus in a private hospital 24 years ago. Her ultrasonogram abdomen revealed features of acute calculus cholecystitis with mild intrahepatic biliary radical dilatation and splenomegaly, for which an open partial cholecystectomy was performed. She was evaluated later for pancytopenia and found to have no obvious hematological etiology. Her family history was insignificant. However, her birth history revealed that it was a domiciliary delivery conducted by a local dai (traditional birth attendant), which could have probably ended in umbilical sepsis. 

On examination, she was oriented, her temperature was 98.2 F, her pulse rate was 78 beats per minute, her respiratory rate was 14 beats per minute, and her oxygen saturation in room air was 97%. Her blood pressure was 112/78 mmHg. There were no indications of icterus, lymphadenopathy, cyanosis, clubbing, edema, or dehydration, nor was the patient pale. Deep palpation of the abdomen indicated mild splenomegaly. The systemic assessment went forward without any notable incidents. The patient was re-evaluated at our hospital for further management. She underwent blood investigations, including liver function tests (LFT), upper gastrointestinal endoscopy (UGI endoscopy), contrast-enhanced computed tomography (CECT), and magnetic resonance imaging (MRI) abdomen (Table 1).

**Table 1:** Laboratory investigations (liver function tests and hematological parameters) ALP: alkaline phosphatase; ALT: alanine transaminase; AST: aspartate aminotransferase; GGT: gamma-glutamyl transpeptidase; HBS-AG: hepatitis B (surface antigen); TC: total count; DC: differential count

Laboratory test	Value	Reference range
Bilirubin total	0.49mg/dL	0.2 -1.2mg/dL
Bilirubin direct	0.16mg/dL	0.0 -0.4 mg/dL
Bilirubin indirect	0.33mg/dL	0.3 – 1.2mg/dL
ALP	66U/L	47 – 118U/L
ALT	12U/L	0-47U/L
AST	22U/L	0-45U/L
GGT	12U/L	0-34U/L
Total protein	7.4g/dl	6.4 -8.2g/dl
Albumin	4.4g/dl	3.4 – 5g/dl
Globulin	3g/dl	2.3 – 3.5g/dl
A/G ratio	1.47	>1
Prothrombin time	12 secs	10.3 – 13.3 secs
INR	0.98	<1.1
Hepatitis C virus	Non-reactive	NA
HBS-AG	Non-reactive	NA
Plasma glucose (fasting)	112mg/dL	70 -110mg/dL
Hemoglobin	9.9g/dL	12 – 15 g/dL
TC	3100cells/cu mm	4000 – 11000cells/cumm
DC – polymorphs	52.8%	45 -70%
Lymphocyte	35.8%	25 -40%
Eosinophil	1.9%	1 – 6%
Monocyte	8.9%	2 – 10%
Basophil	0.6%	0 – 1%
RBC count	3.06mill/ccmm	3.8 – 4.8mill/cumm
Platelet count	1.2lakhs/cumm	1.5 – 4.5lakhs/cumm

**Figure 1:**
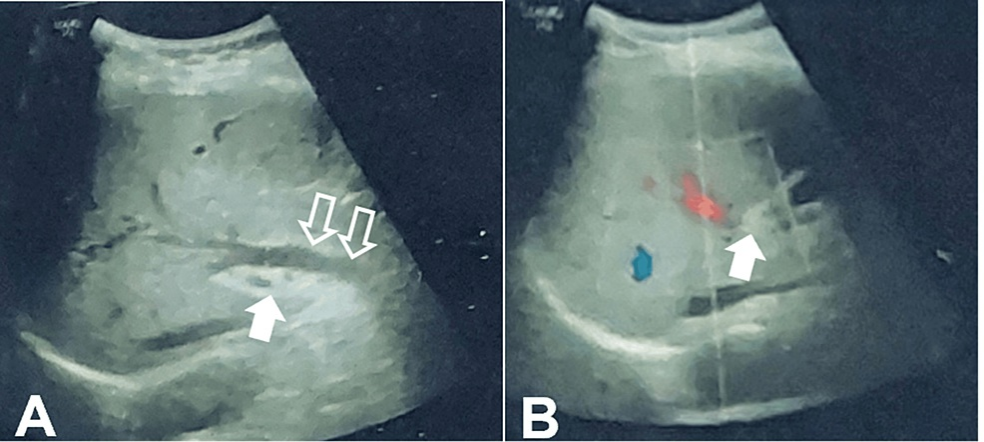
USG findings A: transverse ultrasound section showing the main portal vein replaced by collaterals (arrow), prominent common bile duct seen (open arrows); B: color Doppler shows no flow in the main portal vein (arrow) and flow within the right portal vein

Ultrasonograms of the pelvis and abdomen showed an enlarged spleen (13.6 cm), a portal vein at the porta replaced by collaterals showing low velocity hepatopetal flow max (14 cm/sec), mild central intra-hepatic biliary radical prominence with dilatation of the common hepatic duct, and a proximal common bile duct maximum caliber of 8-9 mm (Figure 1).

**Figure 2:**
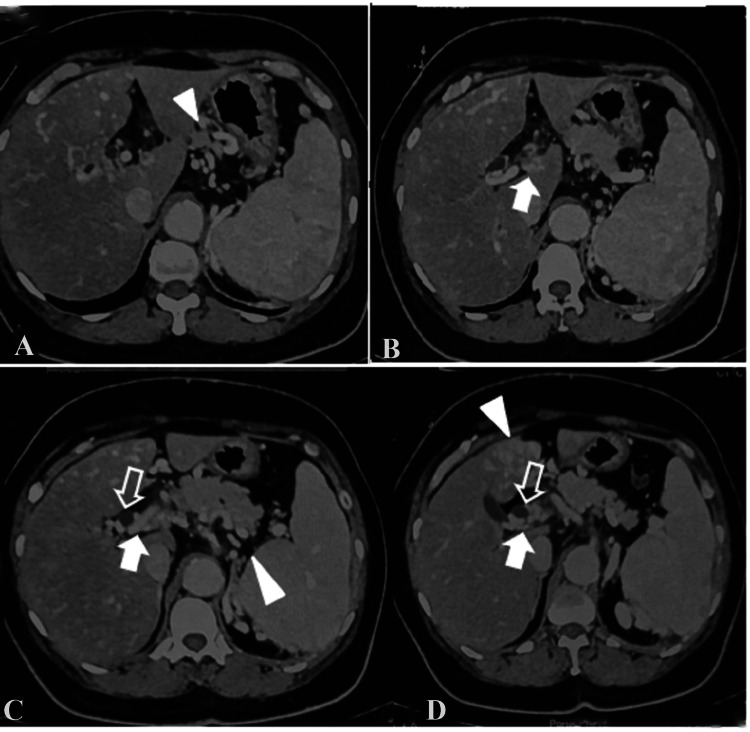
Contrast-enhanced computerized tomography (CECT) findings A-D: axial contrast-enhanced computerized tomography (CECT) shows cavernoma at the portal (arrow), prominent common bile duct (open arrows), and perigastric and peripancreatic collaterals (arrow head)

Her contrast-enhanced computed tomography abdomen and pelvis findings revealed features of chronic extrahepatic portal venous obstruction with normal liver parenchyma. She had asymptomatic portal biliopathy in the form of mild common bile duct narrowing due to portal cavernoma and secondary intrahepatic biliary radical dilatation bilateral (Figure 2).

**Figure 3:**
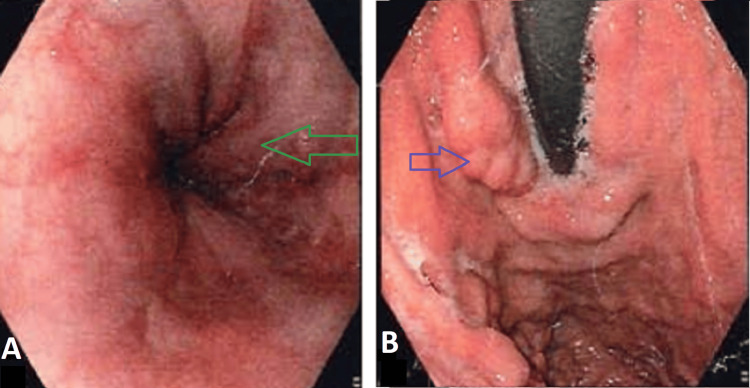
Upper gastrointestinal tract (UGI) scopy findings A: grade IV esophageal varices (green arrow); B: fundal varices - GOV1 (blue arrow) GOV1: gastroesophageal varices

Her liver function tests were normal and were not suggestive of any obstructive pattern. She had large collaterals in the perigastric and gastroepiploic areas, correlating with the endoscopic findings of grade IV esophageal varices, gastroesophageal varices (GOV1), fundal varices, isolated gastric varices (IGV2), and antral varices (Figure 3). 

**Figure 4:**
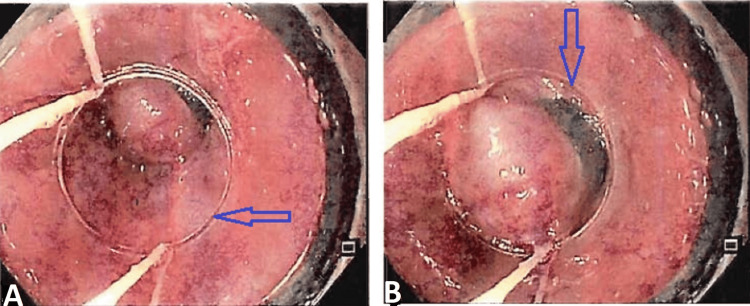
Upper gastrointestinal tract (UGI) scopy during endoscopic banding A, B: endoscopic variceal ligation (blue arrows)

The inferior mesenteric vein was also dilated, with an engorged periuterine plexus, suggesting pelvic congestion syndrome. She was also found to have atherosclerotic major abdominal arteries with chronic proximal superior mesenteric artery thrombosis and collateralization. Her coeliac axis also showed atherosclerosis, but with a patent vessel. After receiving an EHPVO diagnosis, she was treated cautiously with beta blockers (carvedilol 3.125 mg bd) and had endoscopic banding done (Figure 4) as a preventative measure against an upper gastro-intestinal bleed.

**Figure 5:**
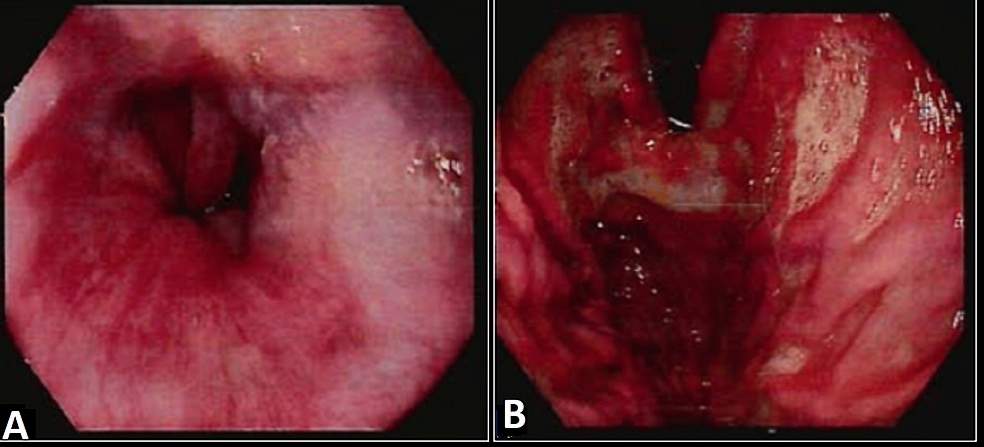
Upper gastrointestinal tract (UGI) scopy showing regression of varices A, B: downgraded esophageal varices

The patient was stable, and a repeat upper gastrointestinal (UGI) scopy was done after six weeks, and regression of varices was observed (Figure 5). The patient is on beta blockers and is advised to review every six months and to report if needed.

## Discussion

EPHVO is the second-most prevalent factor contributing to portal hypertension. Research has shown that symptoms tend to appear in two distinct age groups. Individuals with intra-abdominal infections or unknown causes usually experience symptoms later, around the age of eight. On the other hand, those with an umbilical venous catheter or umbilical sepsis typically develop symptoms earlier, around the age of three [10].

Nevertheless, this patient was asymptomatic but had a medical history indicating a likely infection in the umbilical cord shortly after birth and also had cholecystitis 24 years ago. The patient sought medical assessment due to fatigue and pancytopenia. Research has also indicated that PVT can be asymptomatic and can be diagnosed in the later stage of life, known as the 'chronic' stage. This diagnosis often occurs incidentally during an imaging procedure for unrelated health concerns or when investigating complications related to portal hypertension, such as variceal bleeding, thrombocytopenia, splenomegaly, and occasionally jaundice [11]. Following the occurrence of acute PVT, there is a process of vascular neo-formation that takes place over a period of three to five weeks. This results in the development of a network of collateral arteries, known as 'portal cavernoma', which connect the two patent parts of the blood vessels located proximally and distally to the thrombus. Nevertheless, these collateral vessels are inadequate and fail to restore normal blood flow into the liver, resulting in the development of portal hypertension at a later stage in life [12]. The primary clinical symptoms of persistent PVT/portal cavernoma are problems associated with portal hypertension. Studies have found that the occurrence of cholelithiasis is more frequent in individuals with extrahepatic portal vein obstruction (EHPVO) [13]. However, this could have been overlooked when this patient was being treated for acute calculus cholecystitis at the previous hospital.

The patient presented with grade IV esophageal varices. Research indicates that bleeding in patients with PVT is typically well-tolerated, and the death rate associated with bleeding is significantly lower compared to individuals with cirrhosis. This difference is likely owing to the intact liver function in PVT patients [14]. Moreover, gastroesophageal varices are often of significant size, but gastric varices are more commonly observed in around 30-40% of individuals [1].

The main objective of EHPVO care is to control the acute variceal bleed and then implement secondary prophylaxis. In patients who have recently had EHPVO, the Baveno consensus V guidelines suggest using low molecular weight heparin (LMWH) followed by oral anticoagulants for a minimum of three months. However, if there is a prothrombotic state present, it is recommended to continue anticoagulation medication for a longer duration. In individuals with chronic extrahepatic portal vein obstruction (EHPVO), such as in our specific situation, the agreement among experts does not cover the use of anticoagulant medications, and there is also a lack of supporting data on the effectiveness of transjugular intrahepatic portosystemic shunt (TIPS). However, local thrombolytic treatments are life-saving among patients with acute superior mesenteric vein thrombosis, and endoscopic treatment is advised to prevent the initial occurrence of acute variceal bleeding. In cases of recurring bleeding, beta blockers are just as beneficial for prevention [15].

## Conclusions

Extrahepatic portal venous obstruction, which can result from either portal vein thrombosis or portal cavernoma, can lead to substantial morbidity and death in individuals with or without cirrhosis. However, EHPVO is not frequently recognized, and its treatment poses considerable challenges. There is a dearth of proper guidelines for managing this illness, even though it is a prevalent cause of acute calculus, cholecystitis, and upper gastrointestinal bleeding. A precise diagnosis and a comprehensive treatment strategy are essential in order to decrease the long-term morbidity and mortality linked to it.
